# Supplementary material for: Skeletal muscle derived Musclin protects the heart during pathological overload
Source: Nat Commun. 2022 Jan 10;13:149. doi: 10.1038/s41467-021-27634-5 (PMC8748430; doi:10.1038/s41467-021-27634-5)
Supplement: Supplementary file 1 — Supplementary information [file 41467_2021_27634_MOESM1_ESM.pdf]

Supplementary Information

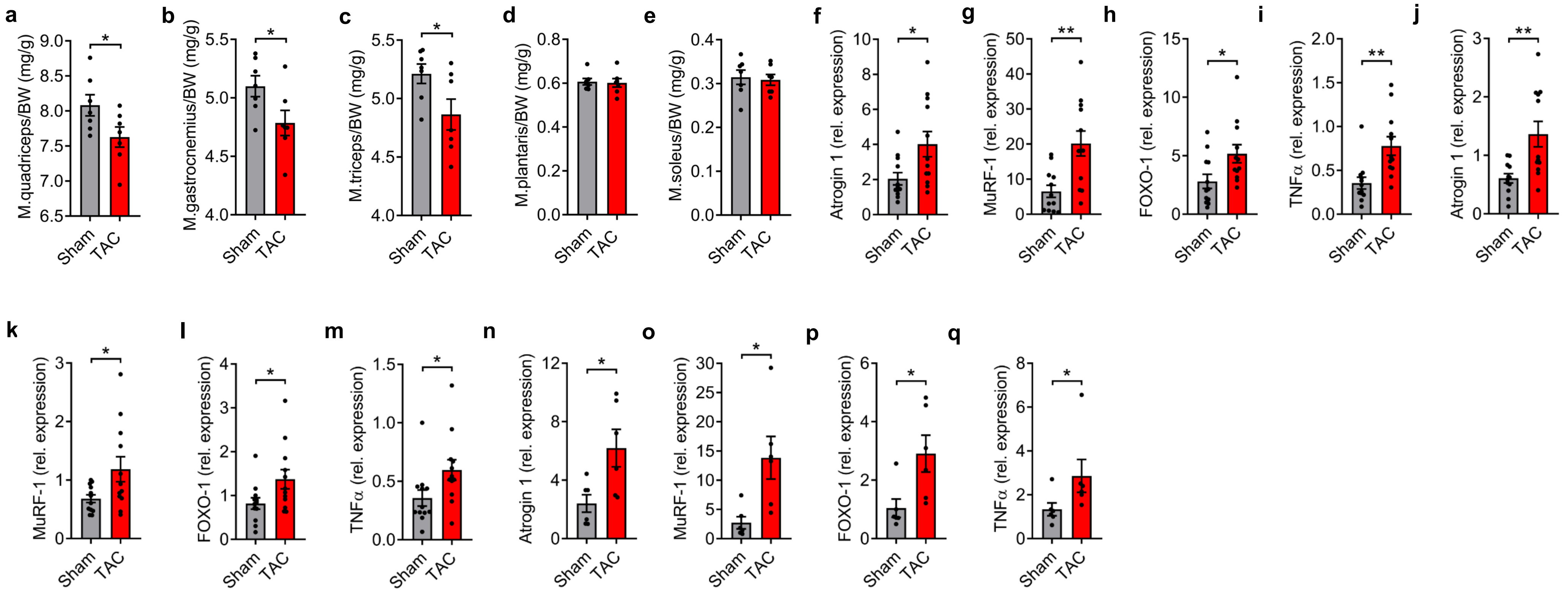

Supplementary Figure 1. Muscle weight normalized to body weight and expression of atrophy and inflammation related genes in skeletal muscle after 12 weeks of TAC. (a-e) Muscle weights of the indicated muscles normalized to body weight (BW) 12 weeks after sham or TAC surgery (n=7 sham and n=7 TAC), *p=0.0496 (a), *p=0.0435 (b), *p=0.0452 (c). **(f-q)** mRNA expression of the indicated genes as determined by qPCR from gastrocnemius muscle (f-i), quadriceps muscle (j-m) and triceps brachii muscle (n-q) 12 weeks after sham or TAC surgery (sham n=12, TAC n=12; except for triceps brachii n=6 for sham and n=6 for TAC), *p=0.021 (f), **p=0.0023 (g), *p=0.0257 (h), **p=0.0027 (i), **p=0.0035 (j), *p=0.0344 (k), *p=0.0414 (l), *p=0.0115 (m), *p=0.0231 (n), *p=0.0146 (o), *p=0.0242 (p), *p=0.0411 (q). Data in bar graphs are shown as mean ± standard error of the mean (SEM). *p<0.05, **p<0.01 as determined by two-tailed Mann–Whitney test (for m and q) or two-tailed Student´s *t*-test (all other numerical data containing panels). Source data are provided as a source data file.

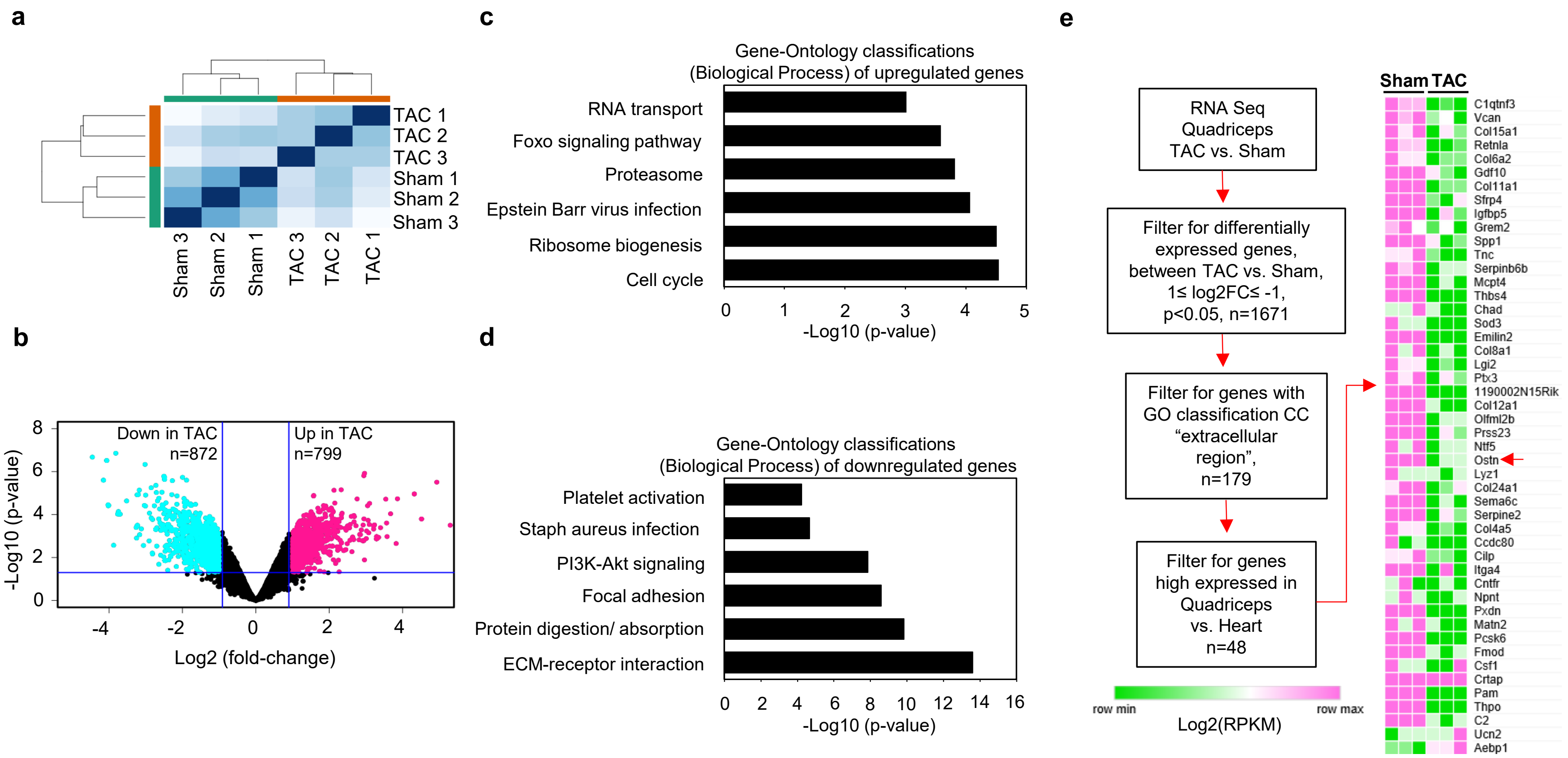

Supplementary Figure 2. Extended results of the RNA Sequencing analysis. (a) Heat map and hierarchical clustering of normalized mapped reads of the RNA sequencing analysis from quadriceps muscles 12 weeks after sham versus TAC (transverse aortic constriction) surgery. **(b)** Volcano plot of the analysis described in (a) with genes significantly up- or downregulated during TAC by at least two-fold. Significance and p-values were calculated by the Wald test. **(c-d)** Gene-Ontology (Biological Process) analysis showing the top six functional categories among upregulated (c) or downregulated gene (d) after TAC. P-values were calculated by modified Fishers exact test. **(e)** Flow chart and heat map demonstrating the identification procedure and the identity of significantly dysregulated skeletal muscle enriched genes encoding for secreted factors from the RNA sequencing (RNA Seq) experiment.

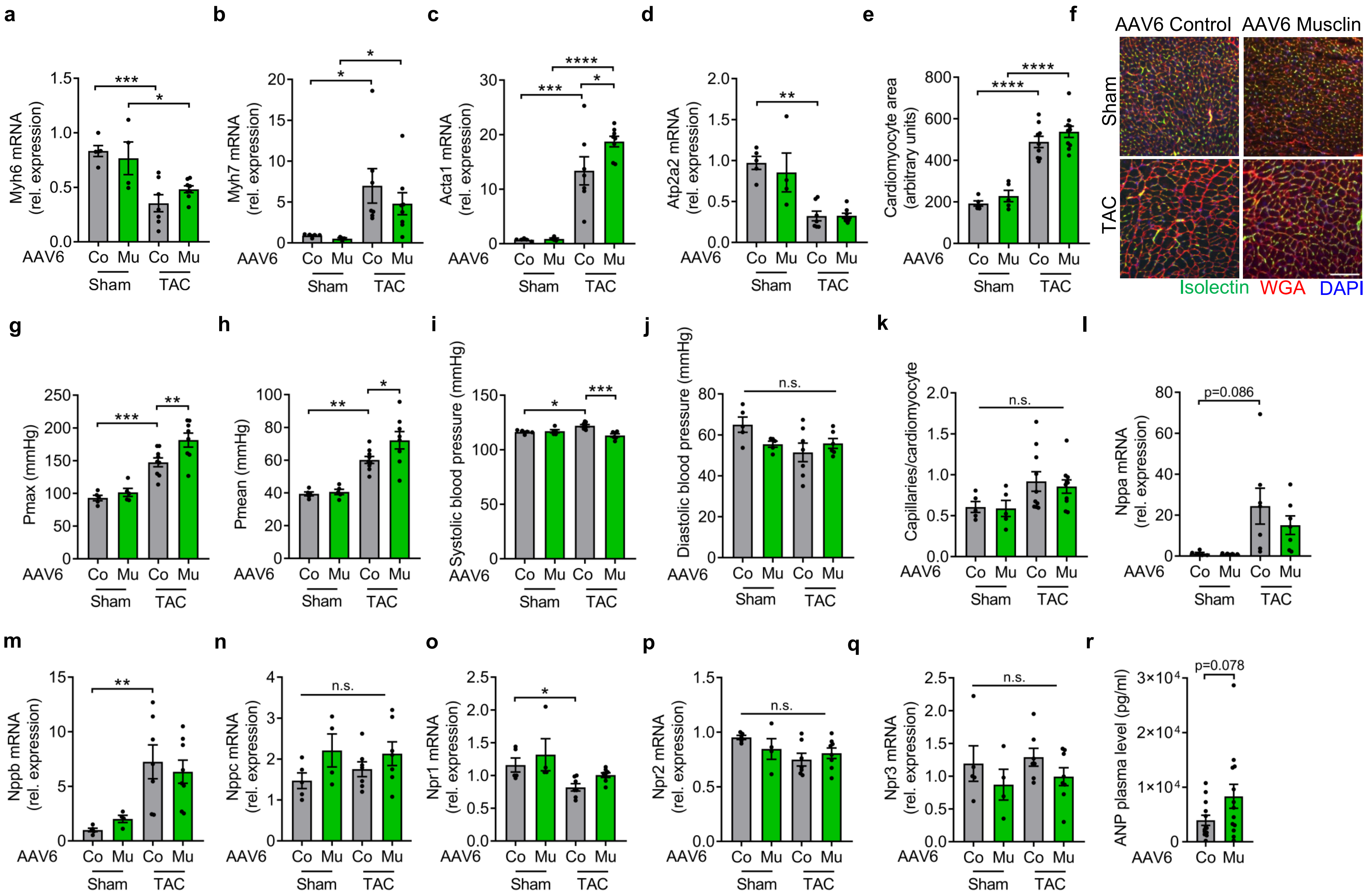

a
Myh6 mRNA (rel. expression)
b
Myh7 mRNA (rel. expression)
c
Acta1 mRNA (rel. expression)
d
Atp2a2 mRNA (rel. expression)
e
Cardiomyocyte area (arbitrary units)
f
AAV6 Control
AAV6 Musclin
Sham
TAC
Isolectin WGA DAPI
g
Pmax (mmHg)
h
Pmean (mmHg)
i
Systolic blood pressure (mmHg)
j
Diastolic blood pressure (mmHg)
k
Capillaries/cardiomyocyte
l
Nppa mRNA (rel. expression)
p=0.086
m
Nppb mRNA (rel. expression)
n
Nppc mRNA (rel. expression)
o
Npr1 mRNA (rel. expression)
p
Npr2 mRNA (rel. expression)
q
Npr3 mRNA (rel. expression)
r
ANP plasma level (pg/ml)
p=0.078
AAV6
Co Mu Co Mu
Sham TAC
n.s.

Supplementary Figure 3. Extended results of morphometric and gene-expression analysis in the experimental groups of AAV6 control (Co) versus AAV6 Musclin (Mu) after sham or TAC surgery. (a-d) qPCR analysis to measure cardiac mRNA levels of indicated hypertrophic marker genes from mice 9 weeks after TAC or sham surgery and treatment with AAV6 Control (Co) or AAV6 Musclin (Mu) (sham AAV6 Co n=5, sham AAV6 Mu n=4, TAC AAV6 Co n=7, TAC AAV6 Mu n=8), ***p=0.0003 for AAV6 Co sham vs. TAC and *p=0.0167 for AAV6 Mu sham vs. TAC (a), *p=0.0164 for AAV6 Co sham vs. TAC and *p=0.0122 for AAV6 Mu sham vs. TAC (b), ***p=0.0001, ****p<0.0001, *p=0.038 (c), **p=0.0025 (d). **(e)** Cardiomyocyte cross-sectional area (sham n=5/group, TAC AAV6 Co n=9, TAC AA6 Mu n=10, ****p<0.0001) and representative immunofluorescence pictures **(f)** stained for Isolectin B4 (to label capillaries) and for WGA (to label cardiomyocyte membranes) used to quantify cardiomyocyte cross-sectional area and capillary density in hearts of the mice described in (a-d). Scale bar: 100µm. **(g)** Systolic maximal, ***p=0.0003 and **p=0.0038 and mean, **p=0.0011 and *p=0.0137 **(h)** left ventricular pressure determined by Millar catheter in the indicated mice (sham n=5/group, TAC AAV6 Co n=9, TAC AAV6 Mu n=8). **(i)** Systolic (*p=0.012, ***p=0.0001) and diastolic **(j)** blood pressure measured by the tail-cuff method in the indicated mice (sham n=5/group, TAC AAV6 Co n=7, TAC AAV6 Mu n=6). **(k)** Capillaries per cardiomyocytes in AAV6 Co versus AAV6 Mu treated mice after sham or TAC surgery (sham n=5/group, TAC n=10/group). **(l-n)** Myocardial mRNA levels of the indicated natriuretic peptides genes (sham AAV6 Co n=5, sham AAV6 Mu n=4, TAC AAV6 Co n=7, TAC AAV6 Mu n=8 except Nppa/Nppc mRNA expression n=7), **p=0.0084 (m). **(o-q)** Myocardial mRNA levels of the indicated natriuretic peptide receptor genes (sham AAV6 Co n=5, sham AAV6 Mu n=4, TAC AAV6 Co n=7, TAC AAV6 Mu n=8), *p=0.0474 (o). **(r)** ANP plasma levels in the indicated mice (n=13). Data in bar graphs are shown as mean ± standard error of the mean (SEM). *p<0.05, **p<0.01, ***p<0.001, ****p<0.0001 determined by two-tailed Student´s *t*-test (r), by Kruskal-Wallis test with Dunn's multiple comparisons test (b, d, o) or one-way ANOVA followed by the Holms-Sidak´s multiple comparisons test (other bar graphs). N.s. denotes "not significant". Source data are provided as a source data file.

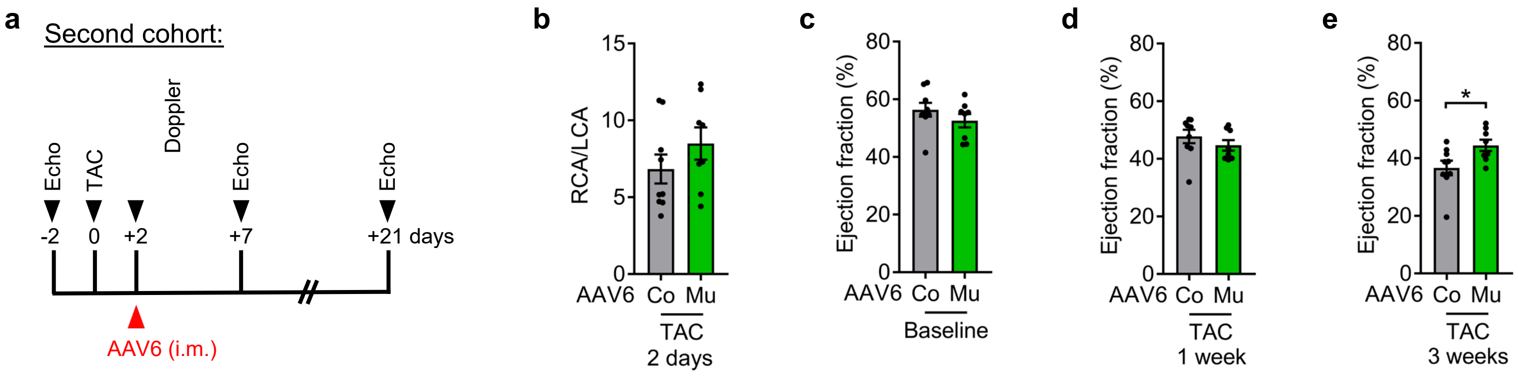

Supplementary Figure 4. Results from the second cohort of mice treated with AAV6 control (Co) versus AAV6 Musclin (Mu) after TAC surgery for 3 weeks (with Doppler examinations and a time course of echocardiographic assessment). (a) Scheme depicting the experimental time line. **(b)** Quantification of Doppler flow velocity ratio of the right common carotid artery (RCA) to left common carotid artery (LCA) 2 days after TAC surgery (for all panels: AAV6-Co n=9, AAV6-Mu n=8). **(c-e)** Echocardiographic determination of ejection fraction in the indicated mice 2 days before TAC surgery (Baseline) **(c)**, as well as 1 week **(d)** and 3 weeks after TAC surgery, *p=0.0309 **(e)**. Data in bar graphs are shown as mean ± standard error of the mean (SEM). *p<0.05 determined by two tailed Student`s *t*-test. Source data are provided as a source data file.

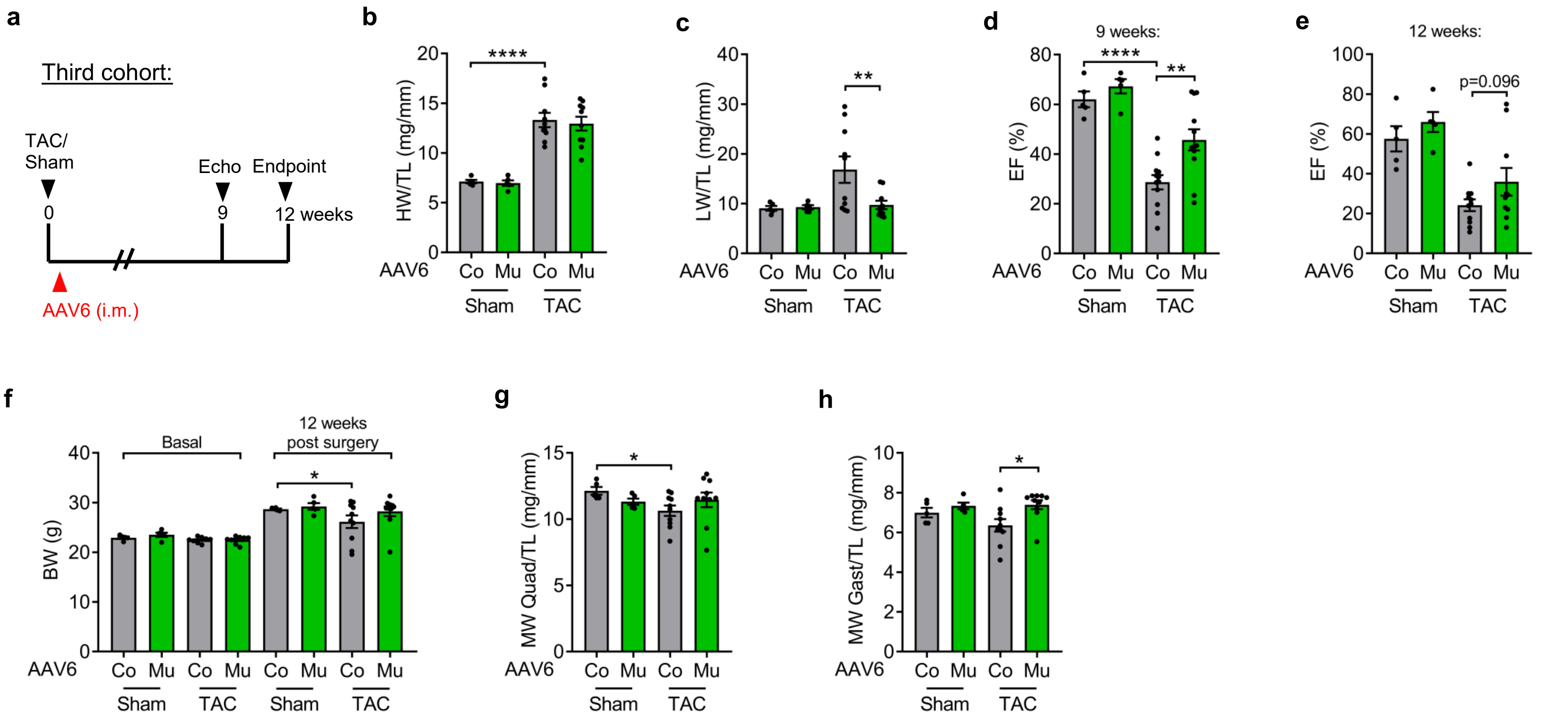

Supplementary Figure 5. Results from the third cohort of mice treated with AAV6 control (Co) versus AAV6 Musclin (Mu) after sham or TAC surgery for 12 weeks (with more morphometric assessments). (a) Scheme depicting the experimental time line. **(b-e)** Quantification of heart weight/tibia length (HW/TL) ratio (b) (sham n=5/group, TAC n=10/group, ****p<0.0001), lung weight/tibia length (LW/TL) ratio (c) (sham n=5/group, TAC n=10/group, **p=0.0052), and left ventricular ejection fraction 9 weeks (d) (sham n=5/group, TAC n=12/group, ****p<0.0001,**p=0.001), and 12 weeks (e) (sham n=5/group, TAC AAV6 Co n=11, TAC AAV6 Mu n=10), after TAC or sham surgery in mice treated with AAV6 control (Co) or AAV6 Musclin (Mu) vector. **(f)** Bodyweight in mice treated as indicated before surgery and 12 weeks after surgery (sham n=5/group, TAC n=10/group, *p=0.0396). **(g-h)** Quantification of quadriceps muscle weight/tibia length (MW Quad/TL) (g), *p=0.0471 and gastrocnemius muscle/tibia length (MW Gast/TL), *p=0.0206 (h) at the end of the experiment 12 weeks after sham or TAC surgery (sham n=5/group, TAC n=10/group). Data in bar graphs are shown as mean ± standard error of the mean (SEM). *p<0.05, **p<0.01, ****p<0.0001 determined by one-way ANOVA followed by the Holms-Sidak´s multiple comparisons test. Source data are provided as a source data file.

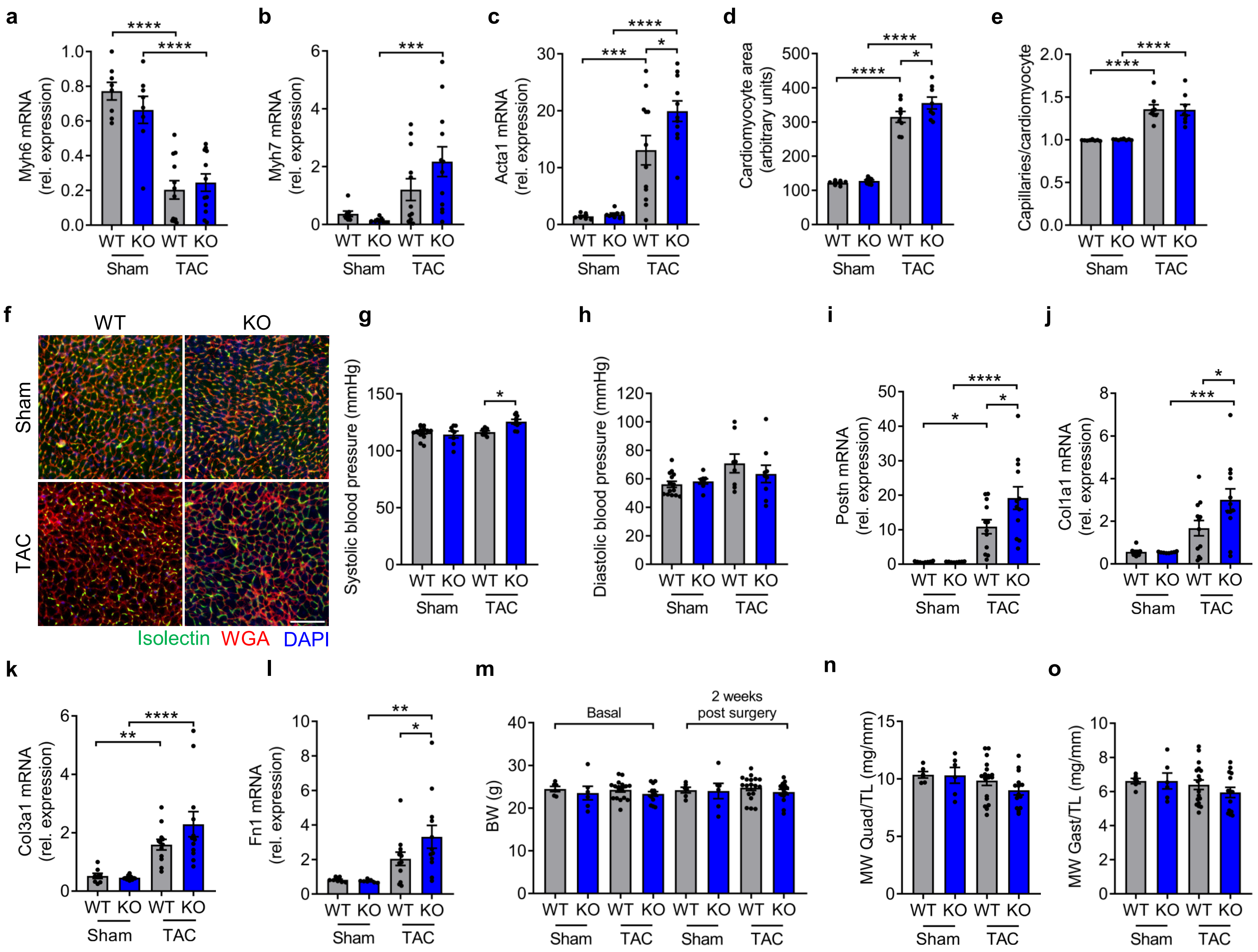
a
Myh6 mRNA (rel. expression)
****
****
1.0
0.8
0.6
0.4
0.2
0.0
WT KO WT KO
Sham TAC
b
Myh7 mRNA (rel. expression)
***
6
4
2
0
WT KO WT KO
Sham TAC
c
Acta1 mRNA (rel. expression)
****
***
*
30
20
10
0
WT KO WT KO
Sham TAC
d
Cardiomyocyte area (arbitrary units)
****
****
*
500
400
300
200
100
0
WT KO WT KO
Sham TAC
e
Capillaries/cardiomyocyte
****
****
2.0
1.5
1.0
0.5
0.0
WT KO WT KO
Sham TAC
f
WT
KO
Sham
TAC
Isolectin WGA DAPI
g
Systolic blood pressure (mmHg)
*
150
100
50
0
WT KO WT KO
Sham TAC
h
Diastolic blood pressure (mmHg)
120
100
80
60
40
20
0
WT KO WT KO
Sham TAC
i
Postn mRNA (rel. expression)
****
*
*
50
40
30
20
10
0
WT KO WT KO
Sham TAC
j
Col1a1 mRNA (rel. expression)
*
***
8
6
4
2
0
WT KO WT KO
Sham TAC
k
Col3a1 mRNA (rel. expression)
****
**
6
4
2
0
WT KO WT KO
Sham TAC
l
Fn1 mRNA (rel. expression)
**
*
10
8
6
4
2
0
WT KO WT KO
Sham TAC
m
BW (g)
Basal
2 weeks post surgery
40
30
20
10
0
WT KO WT KO WT KO WT KO
Sham TAC Sham TAC
n
MW Quad/TL (mg/mm)
15
10
5
0
WT KO WT KO
Sham TAC
o
MW Gast/TL (mg/mm)
10
8
6
4
2
0
WT KO WT KO
Sham TAC

Supplementary Figure 6. Extended results of morphometric and gene-expression analysis in control (WT) versus Musclin knock-out (KO) mice after sham or transverse aortic constriction (TAC) surgery. (a-c) qPCR analysis to measure cardiac mRNA levels of indicated hypertrophic marker genes from the indicated mice 2 weeks after sham or TAC surgery (sham n=8/group, TAC n=12/group except Acta1 mRNA expression TAC KO n=11), ****p<0.0001 (a), ***p=0.0002 (b), ***p=0.0003, *p=0.01 and ****p<0.0001 (c). **(d)** Cardiomyocyte cross-sectional area (n=8/group), myocardial capillary density (n=8/group), ****p<0.0001, *p=0.0468 **(e)** and representative immunofluorescence pictures **(f)** stained for Isolectin B4 (to label capillaries) and for WGA (to label cardiomyocyte membranes) used to quantify both parameters. Scale bar: 100µm **(g)** Systolic and diastolic **(h)** blood pressure measured by the tail-cuff method in the indicated mice (sham WT n=14, sham KO n=8, TAC WT n=8, TAC KO n=9, *p=0.01). **(i-l)** qPCR analysis to measure myocardial mRNA levels of the indicated fibrosis related genes from the indicated mice 2 weeks after sham or TAC surgery (sham n=8/group, TAC n=12/group), *p=0.0138 for WT mice sham vs. TAC, ****p<0.0001 for KO mice sham vs. TAC and *p=0.0179 for WT vs. KO mice after TAC (i), ***p=0.0004 and *p=0.0416 (j), **p=0.0023 and ****p<0.0001 (k), *p=0.0414 and **p=0.001(l). **(m)** Bodyweight of the indicated mice before surgery and 2 weeks after surgery (sham n=6/group, TAC WT n=18, TAC KO n=14). **(n-o)** Quantification of quadriceps muscle weight/tibia length (MW Quad/TL) (g) and gastrocnemius muscle/tibia length (MW Gast/TL) (h) at the end of the experiment 2 weeks after sham or TAC surgery (sham n=6/group, TAC WT n=18, TAC KO n=14). Data in bar graphs are shown as mean ± standard error of the mean (SEM). *p<0.05, **p<0.01, ***p<0.001, ****p<0.0001 determined by Kruskal-Wallis test with Dunn's multiple comparisons test (b and k) or by one-way ANOVA followed by the Holms-Sidak´s multiple comparisons test (all other panels). Source data are provided as a source data file.

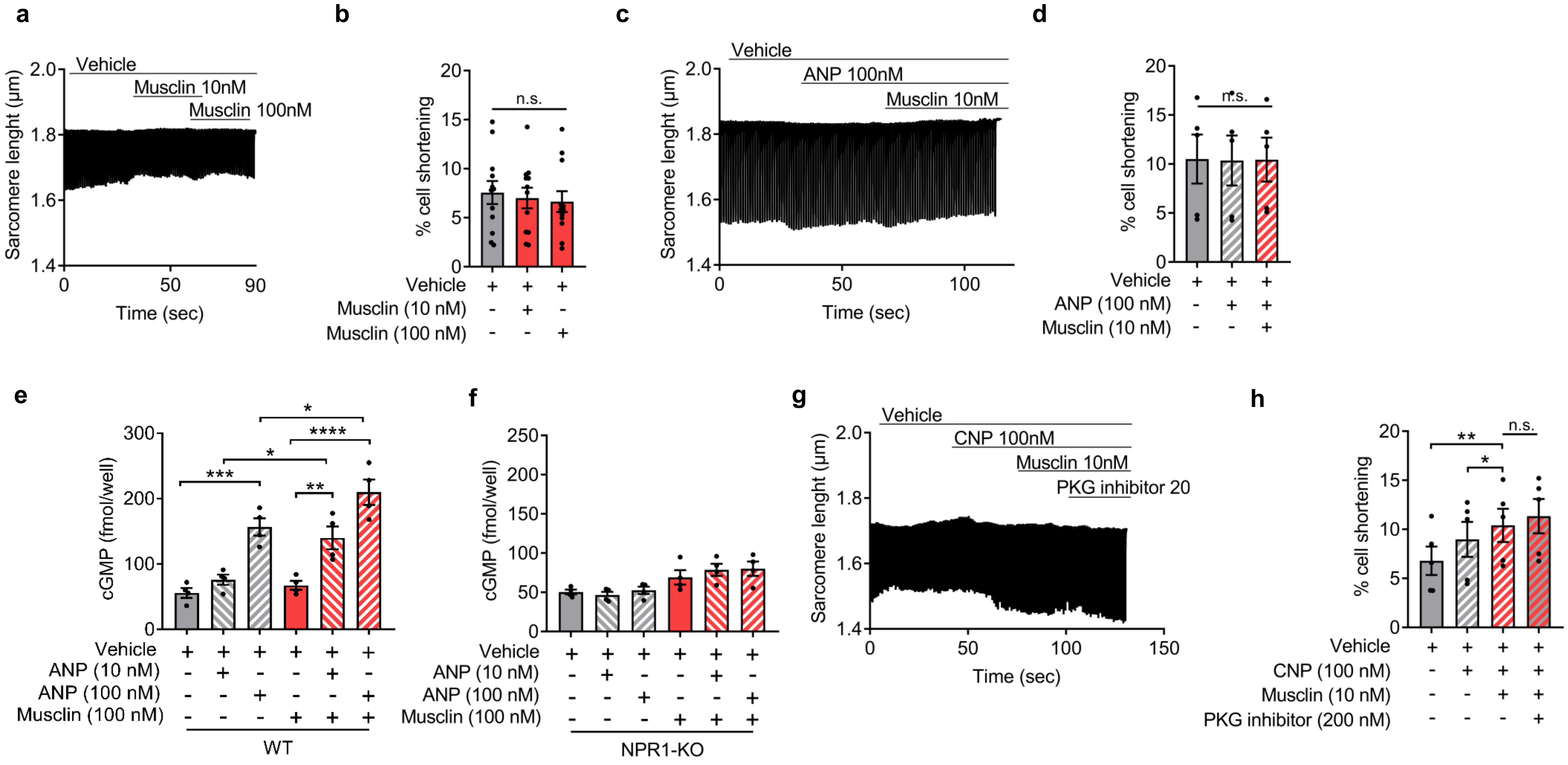

Supplementary Figure 7. Extended results of cardiomyocyte contractility and cGMP measurements. (a-b) Representatives traces of sarcomere length (a) and quantification of cell shortening (from traces as shown in a) in isolated wild-type mouse cardiomyocytes treated with recombinant Musclin as indicated (n=12 cardiomyocytes per condition). **(c)** Representative sarcomere length and quantification of cell shortening **(d)** of wild-type mouse cardiomyocytes treated with recombinant ANP and Musclin as shown (n=5/condition). **(e-f)** ELISA based cGMP measurements in cardiomyocytes from wild-type WT, ***p=0.0002, *p=0.0112 (ANP 10nM without vs. with Musclin), **p=0.0049, *p=0.0299 (ANP10nM without vs. with Musclin), ****p<0.0001 (e), and cardiomyocyte specific Npr1 knock-out (KO) mice (f) (n=4 per condition). **(g-h)** Representative sarcomere length (g) and quantification of cardiomyocyte shortening (h) after treatment with recombinant CNP and Musclin as well as the PKG inhibitor DT3 as indicated (n=5 per condition, **p=0.002 and *p=0.0453). Data in bar graphs are shown as mean ± standard error of the mean (SEM). *p<0.05, **p<0.01, ***p<0.001, ****p<0.0001 determined by one-way ANOVA followed by the Holms-Sidak´s multiple comparisons test. N.s. denotes “not significant”. Source data are provided as a source data file.

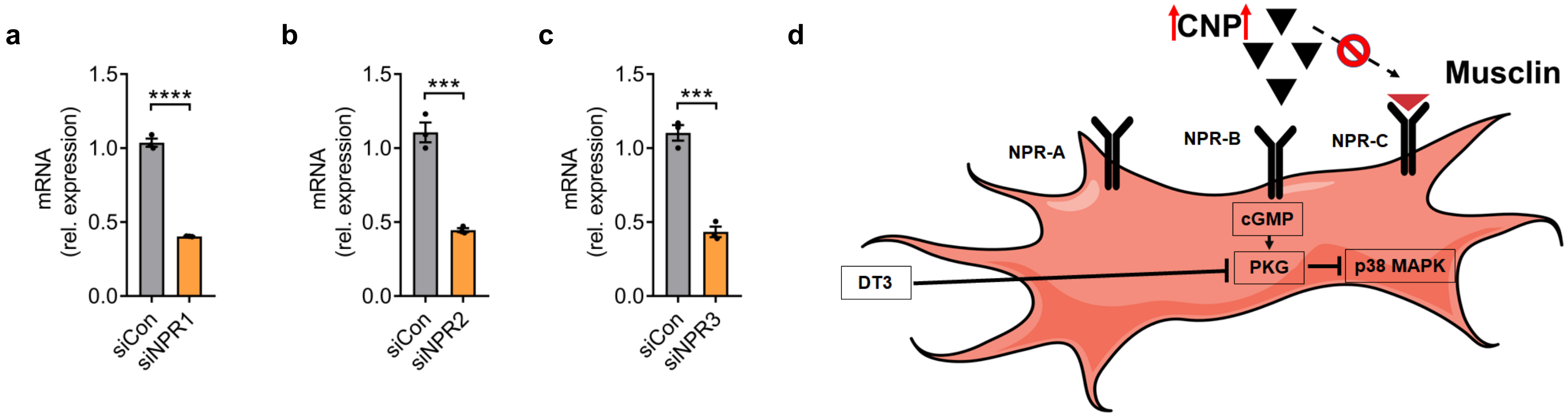

Supplementary Figure 8. Extended results of fibroblast activation assays. (a-c) qPCR analysis of mRNA expression of the indicated genes in cardiac fibroblasts (n=3), ****p<0.0001 (a), ***p=0.0006 (b) and ***p=0.0005 (c). **(d)** Scheme depicting the proposed mechanism of Musclin effects in cardiac fibroblasts. Data in bar graphs are shown as mean ± standard error of the mean (SEM). ***p<0.001, ****p<0.0001 determined by two-tailed Student´s *t*-test. Source data are provided as a source data file.

Supplementary Table 1:

Echocardiographic data after intramuscular AAV6-control (Co) or AAV6-Musclin (Mu) treatment and after Sham or TAC surgery as mean ± SEM.

	Time after Surgery	AAV-Co N=5	AAV-Mu N=5	AAV-Co N=17	AAV-Mu N=15
**Ejection fraction [%]**		65.1±2.2	66.13±1.9	33.1±2.9$^{\#\#, p=0.0015}$	42.9±3.9$^{\#\#\#\#p<0.0001,*p=0.0293}$
**LVEDA [mm]**	3 weeks	10.8±0.5	10±0.5	13.7±1	13.2±0.9
**Wall thickness [mm]**		0.6±0.02	0.7±0.04	0.9±0.04$^{\#\#\#\#p<0.0001}$	0.9±0.03$^{\#p=0.0112}$
**Heart rate [beats per minute]**		571±17	577±9	553±10	570±7
	**Time after Surgery**	**AAV-Co N=5**	**AAV-Mu N=5**	**AAV-Co N=7**	**AAV-Mu N=6**
**Ejection fraction [%]**		68.7±2.1	67.7±4.2	22.71±3.4$^{\#\#\#\#p<0.0001}$	39.4±3.8$^{\#\#\#\#p<0.0001, **p=0.0021}$
**LVEDA [mm]**	6 weeks	10.5±0.6	10.1±0.5	17.8±2.3$^{\#p=0.0207}$	15.2±1.8
**Wall thickness [mm]**		0.6±0.01	0.7±0.03	0.99±0.05$^{\#\#\#\#p<0.0001}$	0.82±0.03$^{\#p=0.0168,**p=0.0059}$
**Heart rate [beats per minute]**		553±14	574±10	535±20	546±17
	**Time after Surgery**	**AAV-Co N=5**	**AAV-Mu N=5**	**AAV-Co N=7**	**AAV-Mu N=6**
**Ejection fraction [%]**		69.2±1.6	65.7±4	20.5±4.9$^{\#\#\#\#p<0.0001}$	38.4±4.4$^{\#\#\#p=0.0006,**p=0.0051}$
**LVEDA [mm]**	9 weeks	10.8±0.6	10.4±0.6	19.2±2.4$^{\#p=0.0112}$	16.1±2.2

**Wall thickness [mm]**		0.65±0.01	0.67±0.04	0.95±0.07$^{\#\#p=0.0032}$	0.94±0.05$^{\#\#p=0.0036}$
**Heart rate [beats per minute]**		528±5.4	543±12	563±19.5	569±12

LVEDA denotes left ventricular enddiastolic area.

$^{\#}p<0.05$, $^{\#\#}p<0.01$, $^{\#\#\#\#}p<0.0001$ vs. the Sham group of the same AAV treatment.

*p<0.05, **p<0.01 vs. AAV-Co TAC; p value was determined by one-way ANOVA followed by the Holms-Sidak´s multiple comparisons test.

Source data are provided as a source data file.

Supplementary Table 2:

Echocardiographic data in control (WT) and skeletal muscle specific Musclin knock-out (KO) mice 2 weeks after Sham or TAC surgery as mean ± SEM.

	**WT Sham N=6**	**KO Sham N=6**	**WT TAC N=18**	**KO TAC N=14**
**Ejection fraction [%]**	62±5	65.4±4	40.4±3[##p=0.0018]	30±2.9[####p<0.0001,*p=0.0445]
**LVEDA [mm]**	11.1±1	10.6±0.4	13.2±0.7	12±0.7
**Wall thickness [mm]**	0.6±0.04	0.6±0.03	0.8±0.03	0.8±0.03
**Heart rate [beats per minute]**	548±8	567±18	551±10	561±14

LVEDA denotes left ventricular enddiastolic area.

[##]p<0.01, [####]p<0.0001 vs. the Sham group of the same genotype

*p<0.05 vs WT TAC; p value was determined by one-way ANOVA followed by the Holms-Sidak´s multiple comparisons test.

Source data are provided as a source data file.

Supplementary Table 3: qPCR Primers

**Gene**	**Sequence 5' - 3'**	**Species**
Npr1-fwd	AAGAGACGATGGGCAGGATA	Mouse
Npr1-rev	CCATCCTCCATGGTGAAGTT	
Npr1-fwd	AAAGATGCCCCGATACTGCC	Rat
Npr1-rev	CATTTCCACATCCCCTCGGA	
Npr2-fwd	ATACCGAGAACCCCCAAATC	Mouse
Npr2-rev	TCTTCGTCCCTGCATCTTCT	
Npr2-fwd	TTGGGGATGCCTACATGGTG	Rat
Npr2-rev	TGCGTAACCTTAGCTGGTCG	
Npr3-fwd	ACACGGAATACTCGCACCTC	Mouse
Npr3-rev	TGATTCTCCGAATGGTGTCA	
Npr3-fwd	TGGAAAAGAAGGCCGGTTCA	Rat
Npr3-rev	TAGGGCCCCCACAACAATTC	
Myh6-fwd	ACTGTGGTGCCTCGTTCC	Mouse
Myh6-rev	GCCTCTAGGCGTTCCTTCTC	
Myh7-fwd	AGGCAAGGCAAAGAAAGGCTCATC	Mouse
Myh7-rev	GCGTGGAGCGCAAGTTTGTCATAA	
Acta1-fwd	GCCAGAGTCAGAGCAGCAGAAACTA	Mouse
Acta1-rev	CAGAGCCGTTGTCACACACAAGA	
Nppa-fwd	ATTGACAGGATTGGAGCCCAGAGT	Mouse
Nppa-rev	TGACACACCACAAGGGCTTAGGAT	
Nppb-fwd	CTCAAGCTGCTTTGGGCACAAGAT	Mouse
Nppb-rev	AGCCAGGAGGTCTTCCTACAACAA	
Nppc-fwd	GCTGGTTCCTGACCAAAAAG	Mouse
Nppc-rev	CAGATGGCTTGTCCAGATCA	
Col1a1-fwd	GACTGGCAACCTCAAGAAGG	Mouse
Col1a1-rev	GGCCAATGTCTAGTCCGAAT	
Col3a1- fwd	ATAAGCCCTGATGGTTCTCG	Mouse
Col3a1-rev	ATGCATGTTTCCCCAGTTTC	
Fn1-fwd	TGACAACTGCCGTAGACCTG	Mouse
Fn1-rev	ATCTAGCGGCATGAAGCACT	
Gapdh-fwd	ACCCAGAAGACTGTGGATGG	Mouse
Gapdh-rev	CACATTGGGGGTAGGAACAC	
Gapdh-fwd	TCGACAGTCAGCCGCATCTTCTTT	Human
Gapdh-rev	ACCAAATCCGTTGACTCCGACCTT	
Ostn-fwd	TGGACTGGAGATTGGCAAGT	Mouse
Ostn-rev	CAGATCATCAAGACGCAGGA	
Ostn-fwd	TTCTCTGGTTTTGGGTCTCCC	Human
Ostn-rev	GCCTCTGGAATTTGAAAGCCG	
Postn-fwd	ACCAGCCAACAAAAGGGTTC	Mouse
Postn-rev	TGCTTGAAGATTGGCTTCTG	
Atp2a2-fwd	ACGTGCCTGGTGGAGAAGATGAAT	Mouse
Atp2a2-rev	ATCTTGCTCATGGATGTCCGGCTT	

**Gene**	**Sequence 5' - 3'**	**Species**
TNFα-fwd	CGAGTGACAAGCCTGTAGCC	Mouse
TNFα-rev	CCTTGTCCCTTGAAGAGAACC	
Atrogin 1-fwd	TACTAAGGAGCGCCATGGATACT	Mouse
Atrogin 1-rev	GTTGAATCTTCTGGAATCCAGGA	
MuRF-1-fwd	GTGTGAGGTGCCTACTTGCT	Mouse
MuRF-1-rev	ACTCAGCTCCTCCTTCACCT	
FOXO-1-fwd	TCAAGGATAAGGGCGACAGC	Mouse
FOXO-1-rev	TGTCCATGGACGCAGCTCTT	

Supplementary Table 4: siRNA sequences

siRNA	Sequence 5' - 3'	Species
ON-TARGET plus SMARTpool siRNA, Npr1	GCAUUGAGUUGACACGAAA<br>GGCUAAAGAUGCCCCGAUA<br>GAAACGUGUGAACCGGAAA<br>CUAGAUUGGGACAGCGAUU	Rat
ON-TARGET plus SMARTpool siRNA, Npr2	CCAGCUAAGGUUACGCAUA<br>CACCAGAAAUUGCUCGAAU<br>CGUGGUGGAUAGUCGAUUU<br>CUGGCUAGCUUCCGAUCAA	Rat
ON-TARGET plus SMARTpool siRNA, Npr3	GCUCUACAGCGACGACAAA<br>CAACAUUGGGAAACAUCGA<br>GGAAAAGAAGGCCGGUUCA<br>AGCACAAGGACACGGAGUA	Rat
