# Supplementary material for: Skeletal muscle derived Musclin protects the heart during pathological overload
Source: Nat Commun. 2022 Jan 10;13:149. doi: 10.1038/s41467-021-27634-5 (PMC8748430; doi:10.1038/s41467-021-27634-5)
Supplement: Supplementary file 3 — Reporting Summary [file 41467_2021_27634_MOESM3_ESM.pdf]

nature research

Corresponding author(s): Joerg Heineke

Last updated by author(s): November 8, 2021

Reporting Summary

Nature Research wishes to improve the reproducibility of the work that we publish. This form provides structure for consistency and transparency in reporting. For further information on Nature Research policies, see Authors & Referees and the Editorial Policy Checklist .

Statistics

For all statistical analyses, confirm that the following items are present in the figure legend, table legend, main text, or Methods section.

n/a	Confirmed	
[ ]	[x]	The exact sample size (*n*) for each experimental group/condition, given as a discrete number and unit of measurement
[x]	[ ]	A statement on whether measurements were taken from distinct samples or whether the same sample was measured repeatedly
[ ]	[x]	The statistical test(s) used AND whether they are one- or two-sided<br>*Only common tests should be described solely by name; describe more complex techniques in the Methods section.*
[x]	[ ]	A description of all covariates tested
[ ]	[x]	A description of any assumptions or corrections, such as tests of normality and adjustment for multiple comparisons
[ ]	[x]	A full description of the statistical parameters including central tendency (e.g. means) or other basic estimates (e.g. regression coefficient) AND variation (e.g. standard deviation) or associated estimates of uncertainty (e.g. confidence intervals)
[ ]	[x]	For null hypothesis testing, the test statistic (e.g. *F*, *t*, *r*) with confidence intervals, effect sizes, degrees of freedom and *P* value noted<br>*Give P values as exact values whenever suitable.*
[x]	[ ]	For Bayesian analysis, information on the choice of priors and Markov chain Monte Carlo settings
[x]	[ ]	For hierarchical and complex designs, identification of the appropriate level for tests and full reporting of outcomes
[x]	[ ]	Estimates of effect sizes (e.g. Cohen's *d*, Pearson's *r*), indicating how they were calculated

Our web collection on statistics for biologists contains articles on many of the points above.

Software and code

Policy information about availability of computer code

Data collection	RNA Sequencing was done by the Illumina HiSeq2500 system; Echo data were collected using VisualSonics Vevo 770 and 3100 systems; Left ventricular pressure was assessed by the PVR-1000, Millar Instruments; Magnetic resonance imaging was obtained using the Bruker, Pharmascan, PHS70/16US system; qPCR was done by the Stratagene MX4000 multiplex qPCR system; Microscopic imaging was done by Zeiss Axioobserver.Z1 and by Leica DMI3000B.
Data analysis	Software used for data analysis: Trim Galore Wrapper tool, version 0.3.5; STAR_2.4.0g1; EdgeR (Version 3.20.5); Adobe Photoshop CS6, Version 6.1; Fiji (ImageJ, Version 2.1.0); Transient Analysis Tool (TAT, Ionoptix, Version 1.7); DAVID tool (Version 6.8); Horos v1.1.7; GraphPad Prism software (version 7.04)

For manuscripts utilizing custom algorithms or software that are central to the research but not yet described in published literature, software must be made available to editors/reviewers. We strongly encourage code deposition in a community repository (e.g. GitHub). See the Nature Research guidelines for submitting code & software for further information.

Data

Policy information about availability of data

All manuscripts must include a data availability statement. This statement should provide the following information, where applicable:

- Accession codes, unique identifiers, or web links for publicly available datasets
- A list of figures that have associated raw data
- A description of any restrictions on data availability

All data presented in this study are available in the main text, the main figures or in the supplementary materials. RNA-seq data generated during this study are available in the Gene Expression Omnibus (GEO) repository (http://www.ncbi.nlm.nih.gov/geo/) and are accessible through GEO series accession number GSE129205 (https://www.ncbi.nlm.nih.gov/geo/query/acc.cgi?acc=GSE129205) . The following genome assembly was used for alignment: mm10, GRCm38 - mm10 - Genome - Assembly - NCBI (nih.gov) . Source data are provided with this paper.

Field-specific reporting

Please select the one below that is the best fit for your research. If you are not sure, read the appropriate sections before making your selection.

- [x] Life sciences
- [ ] Behavioural & social sciences
- [ ] Ecological, evolutionary & environmental sciences

For a reference copy of the document with all sections, see nature.com/documents/nr-reporting-summary-flat.pdf

Life sciences study design

All studies must disclose on these points even when the disclosure is negative.

Sample size	Sample size was chosen as a result of previous experience regarding data variability in similar models and experimental set-ups (66, 67, 75, 76). No statistical method was used to predetermine sample size. (page 31)
Data exclusions	Premature death was a criterion for exclusion from an ongoing experiment. (page 22)
Replication	All experiments were carried out in at least 3 biological replicates. The exact number of biological replicates (number of mice, samples or cell culture dishes) is mentioned in the Figure legends. (page 31)
Randomization	Mice were allocated to the different experimental group due to genotyping results. Wild-type mice were randomly asigned to receive AAV6 Control or AAV6 Musclin treatment. Healthy individuals, patients suffering from severe aortic stenosis or from chronic heart failure with cachexia or sarcopenia were assigned to the experimental groups primarily as result of their diagnosis (or due to absence of disease for healthy individuals). For the serum analysis all samples (randomly collected during between May 2011 and February 2014 among patient with severe aortic stenosis or from blood donors) available in our lab were analyzed and included in the manuscript. For the skeletal muscle study, samples in each group were randomly chosen from the existing samples of the group. (page 31/32)
Blinding	The investigators were blinded for mouse genotype and treatment during surgeries, echocardiography, cardiac catheterization, organ weight determination and all histological and immunofluorescence quantifications. For other experiments, researchers were not blinded to group allocation during data collection due to the neccesity of knowing the treatment to be administered or the samples to be collected. However, researchers were blinded to group allocation during data analysis in most of the cases. (page 32)

Reporting for specific materials, systems and methods

We require information from authors about some types of materials, experimental systems and methods used in many studies. Here, indicate whether each material, system or method listed is relevant to your study. If you are not sure if a list item applies to your research, read the appropriate section before selecting a response.

Materials & experimental systems

n/a	Involved in the study	
[ ]	[x]	Antibodies
[ ]	[x]	Eukaryotic cell lines
[x]	[ ]	Palaeontology
[ ]	[x]	Animals and other organisms
[ ]	[x]	Human research participants
[x]	[ ]	Clinical data

Methods

n/a	Involved in the study	
[x]	[ ]	ChIP-seq
[x]	[ ]	Flow cytometry
[x]	[ ]	MRI-based neuroimaging

Antibodies

Antibodies used	Musclin (#RD181079100, 1µg/ml, BioVendor), Actin (#A2066, 1:100, Sigma), alpha Tubulin (#ab40742, 1:5000, Abcam) and Serca2a ATPase (#ab2861, 1:1000, Abcam), Phospholamban (#A010-14, 1:2000, Badrilla) and Phospholamban (pSer16) (#A010-12, 1:5000, Badrilla), p38 MAPK (#9212, 1:1000, Cell Signaling), Phospho-p38 MAPK (T180/Y182), (#9211, 1:1000, Cell Signaling), p44/42 MAPK (Erk1/2), (#9102, 1:1000, Cell Signaling), Phospho-p44/42 MAPK (T202/Y204), (#9101, 1:1000, Cell Signaling). The blots were hybridized with an antibody against GAPDH (#10R-G109a, 1:6000, Fitzgerald) to verify equal loading of protein in each lane. The following secondary antibodies were used: Anti-Rabbit-IgG-HRP (#NA934, 1:3500, GE Healthcare Life Sciences), anti-Mouse-IgG-HRP (#NXA931, 1:3500, GE Healthcare Life Sciences) and anti-Goat-IgG-HRP (#HAF005, 1:10000, R&D systems).(page 26)
Validation	The Musclin antibody from the German company Biovendor detected a band at the expected size (at about 15kDa), which was enhanced after Musclin overexpression and absent in the heart (where Musclin is not expressed) or in Musclin knock-out mice, where Musclin levels were strongly reduced (see Figures 3c and 4e). All other antibodies were widely used before. e.g. Heineke J et al., 2010, Nature Medicine; Zwadlo C et al., 2015, Circulation; Frantz S et al., 2013, Eur Heart J

Eukaryotic cell lines

Policy information about cell lines

Cell line source(s)	Cell Line 293 ACC 305 (HEK293) from Leibniz Institute DSMZ
Authentication	The cell line was not authenticated.
Mycoplasma contamination	The cells were not tested for Mycoplasma infection.
Commonly misidentified lines (See ICLAC register)	No commonly misidentified cell lines were used in this study.

Animals and other organisms

Policy information about studies involving animals; ARRIVE guidelines recommended for reporting animal research

Laboratory animals	Male C57Bl/6JCrl mice (Charles River Laboratories) at 8-10 weeks of age were used for all experiments (except for the experiments involving Musclin KO and littermate WT mice, as well as Npr1 and Npr2 knock-out and corresponding wild-type mice, where male and female mice were used). The animals had free access to water and a standard diet and were maintained on a 12-h light and dark cycle at a room temperature of 22 ± 2°C and a humidity of 35-60%. TAC surgery was performed by subjecting the aorta to a defined 27-gauge constriction in 8-10 weeks old mice as described previously (66, 67). Cardiomyocyte specific, 8-10 weeks old Npr1 (NPR-A) knock-out mice and global Npr2 (NPR-B) knock-out mice were previously published (68, 69). (page 21)<br>For experiments, Doxycycline (Sigma Aldrich) was dissolved in drinking water at a concentration of 2mg/ml and given to animals in light-protected water bottles for one week before TAC or sham surgery. Littermate male and female Ostnfl/fl mice (Musclin WT) on a C57Bl/6J background as control and Ostnfl/fl (HSA-rtTA/TRE)Cre mice (Musclin KO), aged between 8-10 weeks, all treated with Doxycycline were included in this study. (page 24)
Wild animals	This study did not involve wild animals.
Field-collected samples	This study did not involve field-collected samples.
Ethics oversight	All procedures involving the use and care of animals were performed according to the Directive 2010/63/EU of the European Parliament on the protection of animals used for scientific purposes and the German animal protection code. Approval was granted by the local state authorities (LAVES-Niedersächsisches Landesamt für Verbraucherschutz und Lebensmittelsicherheit, 33.14-42502-04-11/0335 and 33.8-42502-04-16/2356). (page 21)

Note that full information on the approval of the study protocol must also be provided in the manuscript.

Human research participants

Policy information about studies involving human research participants

Population characteristics	Characteristics of the study population and the healthy volunteers are described in detail in Table 1 and Table 2
Recruitment	Muscle biopsies of the vastus lateralis muscle were obtained from control subjects without history of cardiovascular or neuromuscular disorders. They were enrolled at the Department of Cardiology, Charité Medical School, Campus Virchow-Klinikum, Berlin, Germany, and the Space Clinic of the Institute of Space Medicine and Physiology (Medes-IMPS, Rangueil Hospital) in Toulouse, France. Sarcopenic and cachectic patients were enrolled in the Studies Investigating Co-morbidities Aggravating Heart Failure (SICA-HF) trial and were enrolled at the Department of Cardiology, Charité Medical School, Campus Virchow-Klinikum, Berlin, Germany. For the studying Musclin in serum, healthy volunteers were blood donors at the Hannover Medical School. The patients were recruited on the Cardiology ward at Hannover Medical School, Germany. Due to prospective enrollment of patients in SICA-HF, when the outcome was not yet clear, self-selection bias is unlikely. Self-selection bias could have affected our serum analysis, but because we anticipate more active patients would enroll (which could lead to higher Musclin levels, see reverence #13, Subbotina E at al., 2015), this would then underestimate the reduction in serum Musclin concentration in patients, which we observed.
Ethics oversight	Muscle biopsy studies:<br>Individuals enrolled at the Institute of Space Medicine and Physiology received a compensation for their participation. All subjects provided written and informed consent before being enrolled. The local ethics committees at the Charite Medical School, Germany, and at the CPP Sud-Ouest et Outre-Mer I, France, approved the studies. The study fulfills all principles of the Declaration of Helsinki. (page 30)<br><br>Serum studies:<br>This study was approved by the Ethical Committee of the Hannover Medical School, Germany. All individuals and patients gave written informed consent. (page 30)

Note that full information on the approval of the study protocol must also be provided in the manuscript.
